# The Scholarly Knowledge Ecosystem: Challenges and Opportunities for the Field of Information

**DOI:** 10.3389/frma.2021.751553

**Published:** 2022-01-31

**Authors:** Micah Altman, Philip N. Cohen

**Affiliations:** ^1^Center for Research in Equitable and Open Scholarship, MIT Libraries, Massachusetts Institute of Technology, Cambridge, MA, United States; ^2^Department of Sociology, University of Maryland, College Park, MD, United States

**Keywords:** scholarly communications, research ethics, scientometrics, open access, open science

## Abstract

The scholarly knowledge ecosystem presents an outstanding exemplar of the challenges of understanding, improving, and governing information ecosystems at scale. This article draws upon significant reports on aspects of the ecosystem to characterize the most important research challenges and promising potential approaches. The focus of this review article is the fundamental scientific research challenges related to developing a better understanding of the scholarly knowledge ecosystem. Across a range of disciplines, we identify reports that are conceived broadly, published recently, and written collectively. We extract the critical research questions, summarize these using quantitative text analysis, and use this quantitative analysis to inform a qualitative synthesis. Three broad themes emerge from this analysis: the need for multi-sectoral cooperation and coordination, for mixed methods analysis at multiple levels, and interdisciplinary collaboration. Further, we draw attention to an emerging consensus that scientific research in this area should by a set of core human values.

## The Growing Importance of the Scientific Information Ecosystem

“The greatest obstacle to discovery is not ignorance—it is the illusion of knowledge.”

—Daniel J. Boorstin

Over the last two decades, the creation, discovery, and use of digital information objects have become increasingly important to all sectors of society. And concerns over global scientific information production, discovery, and use reached a fever-pitch in the COVID-19 pandemic, as the life-and-death need to generate and consume scientific information on an emergency basis raised issues ranging from cost and access to credibility.

Both policymakers and the public at large are making increasingly urgent demands to understand, improve, and govern the large-scale technical and human systems that drive digital information. The scholarly knowledge ecosystem^1^ presents an outstanding exemplar of the challenges of understanding, improving, and governing information ecosystems at scale.

Scientific study of the scholarly knowledge ecosystem has been complicated by the fact that the topic is not the province of a specific field or discipline. Key research in this area is scattered across many fields and publication venues. This article integrates recent reports from multiple disciplines to characterize the most significant research problems—particularly grand challenges problems—that pose a barrier to the scientific understanding of the scholarly research ecosystem, and traces the contours of the approaches that are most broadly applicable across these grand challenges.^2^

The remainder of the article proceeds as follows: Characterizing the Scholarly Knowledge Ecosystem section describes our bibliographic review approach and identifies the most significant reports summarizing the scholarly knowledge ecosystem. Embedding Research Values section summarizes the growing importance of scientific information and the emerging recognition of an imperative to align the design and function of scholarly knowledge production and dissemination with societal values. Scholarly Knowledge Ecosystem Research Challenges section characterizes—impact scientific research problems selected from these reports. Commonalities Across the Recommended Solution Approaches to Core Scientific Questions section identifies the common shared elements of solution approaches to these scientific research problems. Finally, Summary section summarizes and comments on the opportunities and strategies for library and information science researchers to engage in new research configurations.

## Characterizing the Scholarly Knowledge Ecosystem

The present and future of research—and scholarly communications—is “more.” By some accounts, scientific publication output has doubled every 9 years, with one analysis stretching back to 1650 (Bornmann and Mutz, 2015). This growth has been accompanied by an increasing variety of scholarly outputs and dissemination channels, ranging from nanopublications to overlay journals to preprints to massive dynamic community databases.^3^ As its volume has multiplied, we have also witnessed public controversies over the scholarly record and its application. These include intense scrutiny of climate change models (Björnberg et al., 2017), questions about the reliability of the entire field of forensic science (National Research Council, 2009), the recognition of social biases embedded in algorithms (Obermeyer et al., 2019; Sun et al., 2019), and the widespread replication failures across medical (Leek and Jager, 2017) and behavioral (Camerer et al., 2018) sciences.

The COVID-pandemic has recently provided a stress test for scholarly communication, exposing systemic issues of volume, speed, and reliability, as well as ethical concerns over access to research (Tavernier, 2020). In the face of the global crisis, the relatively slow pace of journal publication has spurred the publication of tens of thousands of preprints (Fraser et al., 2020), which in turn generated consternation over their veracity (Callaway, 2020) and the propriety of reporting on them in major news media (Tingley, 2020).

This controversy underscores calls from inside and outside the academy to reexamine, revamp, or entirely re-engineer the systems of scholarly knowledge creation, dissemination, and discovery. This challenge is critically important and fraught with unintended consequences. While calls for change reverberate with claims such as “taxpayer-funded research should be open,” “peer review is broken,” and “information wants to be free,” the realities of scholarly knowledge creation and access are complex. Moreover, the ecosystem is under unprecedented stress due to technological acceleration, the disruption of information economies, and the divisive politics around “objective” knowledge. Understanding large information ecosystems in general and the scientific information ecosystem in particular, presents profound research challenges with huge potential societal and intellectual impacts. These challenges are a natural subject of study for the field of information science. As it turns out, however, much of the relevant research on scholarly knowledge ecosystems is spread across a spectrum of other scientific, engineering, design, and policy communities outside the field of information.

We aimed to present a review that is useful for researchers in the field of information in developing and refining research agendas and as a summary for regulators and funders of areas where research is most needed. To this end, we sought publications that met the following three criteria:

- *Broad*
  ° Characterizing a broad set of theoretical, engineering, and design questions relevant to how people, systems, and environments create, access, use, curate, and sustain scholarly knowledge.
  ° Covering multiple research topics within scholarly knowledge ecosystems.
  ° Synthesizing multiple independent research findings.

- *Current*
  ° Indicative of current trends in scholarship and scholarly communications.
  ° Published within the last 5 years, with substantial coverage of recent research and events.

- *Collective*
  ° Reflecting the viewpoint of a broad set of scholars.
  ° Created, sponsored, or endorsed by major research funders or scholarly societies.
  ° Or published in a highly visible peer-reviewed outlet.

To construct this review, we conducted systematic bibliographic searches across scholarly indices and preprint archives. This search was supplemented by forward- and backward- citation analysis of highly cited articles; and a systematic review of reports from disciplinary and academic societies. We then filtered publications to operationalize the selection goals described above. This selection process yielded the set of eight reports, listed in Table 1.

**Table 1:** Key reports relevant to the scholarly knowledge ecosystem.

**Year**	**Title**	**Description**	**Citation/References**
2020	NDSA agenda for Digital Stewardship	Community/expert synthesis report conducted through *National Digital Stewardship Alliance*	(NDSA, 2020) (Digital stewardship)
2020	Calibrating the scientific ecosystem through meta-research	Scientific review published in *Annual Review of Statistics and Its Application*	(Hardwicke et al., 2020) (Meta research)
2019	The global landscape of AI ethics guidelines	PRISMA-review of AI ethics principles from 84 large organizations, societies, governments	(Jobin et al., 2019) (AI ethics)
2019	Reproducibility and replicability in science	Expert consensus report on reproducibility, convened by National Academies Committee on Reproducibility and Reliability	(NASEM-BCBSS, 2019) (reproducibility)
2018	A Grand Challenges-Based Research Agenda for Scholarly Communication and Information Science	Community-based synthesis report convened by MIT Center for Research on Equitable and Open Scholarship and Mellon Foundation	(Altman et al., 2018) (grand challenges)
2019	Open and Equitable Scholarly Communications: Creating a More Inclusive Future	Community-based synthesis report convened by Association of College and Research Libraries	(Maron et al., 2019) (SCHOLCOM)
2018	Open science by design: Realizing a vision for 21st-century research	Expert consensus report on open science convened by National Academies Board on Research Data and Information.	(NASEM–BRDI, 2018) (Open SCI)
2016	Ethically aligned design	Community/expert synthesis report convened by Institute of Electrical and Electronics Engineers	(Leek and Jager, 2017) (EAD)

Collectively the reports in Table 1 integrate perspectives from scores of experts, based on examination of over one thousand research publications and scholarship from over a dozen fields. In total, these reports span the primary research questions associated with understanding, governing, and reengineering the scholarly knowledge ecosystem.

To aid in identifying commonalities across these reports, we coded each report to identify important research questions, broad research areas (generally labeled as opportunities or challenges), and statements declaring core values or principles needed to guide research. We then constructed a database by extracting the statements, de-duplicating them (within work), standardizing formatting, and annotating them for context.^4^ Table 2 summarizes the number of unique coded statements in each category by type and work.

**Table 2:** Extent of coded content.

**Work**	**Research questions**	**Research areas**	**Values**	**Total**
AI ETHICS	0	1	11	12
DIGITAL STEWARDSHIP	0	7	4	11
EAD	7	3	8	18
GRAND CHALLENGES	32	6	5	43
META RESEARCH	0	4	2	6
OPEN SCI	5	5	2	12
REPRODUCIBILITY	3	3	3	9
SCHOLCOM	0	18	3	21

## Embedding Research Values

Science and scholarship have played a critical role in the dramatic changes in the human condition over the last three centuries. The scientific information ecosystem and its governance are now recognized as essential to how well science works and for whom. Without rehearsing a case for the value of science itself, we observe that the realization of such value is dependent on a system of scholarly knowledge communication.

In recent years we have seen that the system for disseminating scholarly communications (including evaluation, production, and distribution) is itself a massive undertaking, involving some of the most powerful economic and political actors in modern society. The values, implicit and explicit, embodied in that system of science practice and communication are vital to both the quality and quantity of its impact. If managing science information is essential to the potential positive effects of science, then the values that govern that ecosystem are essential building blocks toward that end. The reports illustrate how these values emerge through a counter-discourse, the contours of which are visible across fields.

All of the reports underscored^5^ the importance of critical values and principles for successful governance of the scholarly ecosystem and for the goals and conduct of scientific research itself. ^6^ These values overlapped but were neither identical in labeling nor substance, as illustrated in Table 3.

**Table 3:** Core values and principles identified in each report.

**Work**	**Values implicated**
AI ETHICS	Transparency; justice, fairness, and equity; non-maleficence; responsibility; privacy; beneficence; freedom and autonomy; trust; sustainability; dignity; solidarity
DIGITAL STEWARDSHIP	Information ethics and privacy; trustworthiness; (organizational) sustainability; environmental sustainability
EAD	Universal human values (well-being); political self-determination and data agency; technical dependability; effectiveness; transparency; accountability; awareness of misuse; competence
GRAND CHALLENGES	Inclusion; openness; social equity; (organizational) sustainability; durability
META RESEARCH	Transparency; reproducibility
Open SCI	Openness; transparency
REPRODUCIBILITY	Science is a communal enterprise; science aims for refined degrees of confidence; scientific knowledge is durable and mutable
SCHOLCOM	Openness; inclusion; social equity

**Figure 1:**
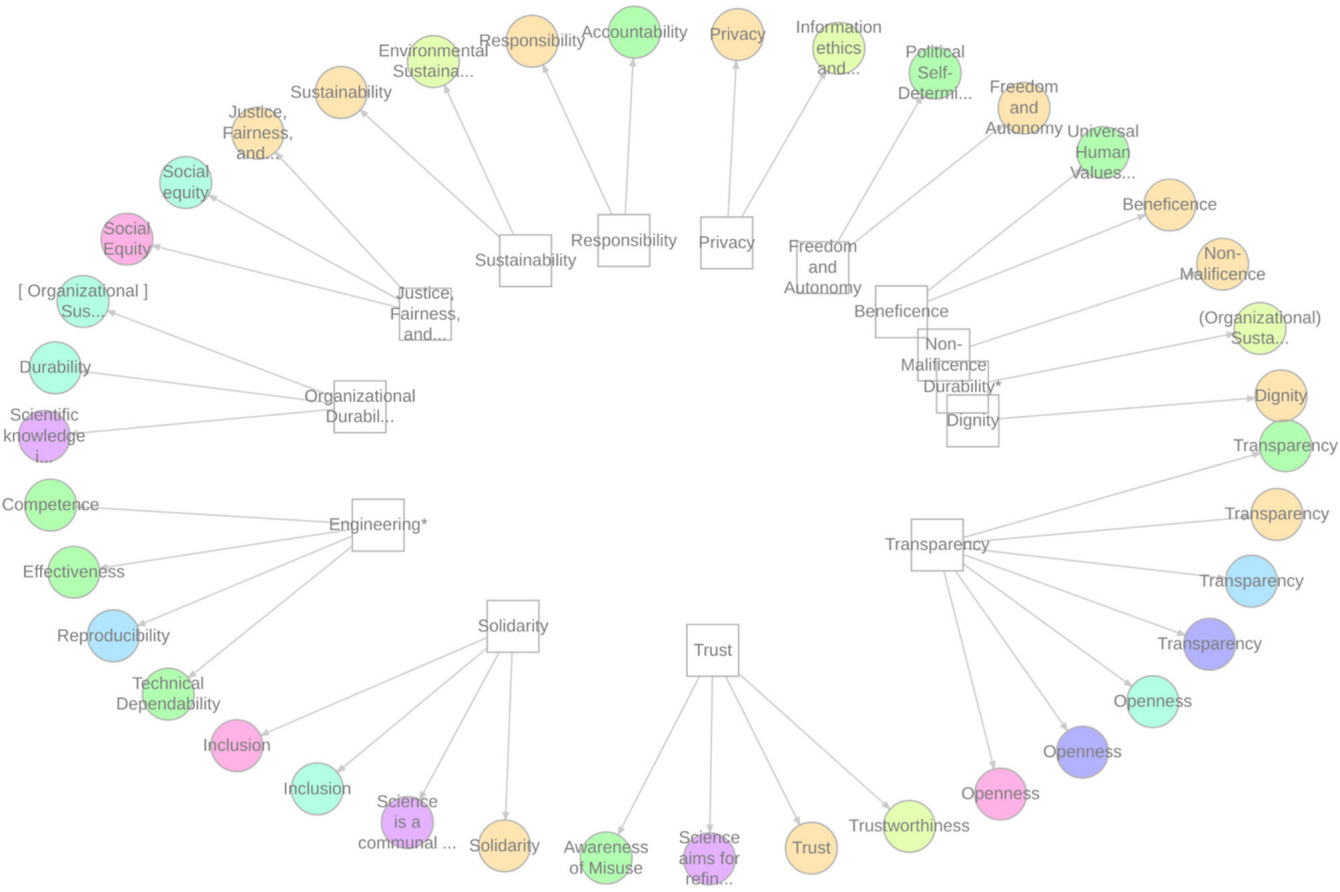
Relationship among values. ^*^Denotes an extension to the core categorization developed in Jobin et al. (2019).

Although the reports each tended to articulate core values using somewhat different terminology, many of these terms referred to the same general normative concepts. To characterize the similarities and differences across reports, we applied the 12-part taxonomy developed by AIETHICS in their analysis of ethics statements to each of the reports. As shown in Figure 1, these 12 categories were sufficient to match almost all of the core principles across reports, with two exceptions: several reports advocated for the value of organizational or institutional sustainability, as distinct from the environmental sustainability category; And the EAD referenced a number of principles, such as “competence” and (technical) “dependability” that generally referred to the value of sound engineering.

**Figure 2:**
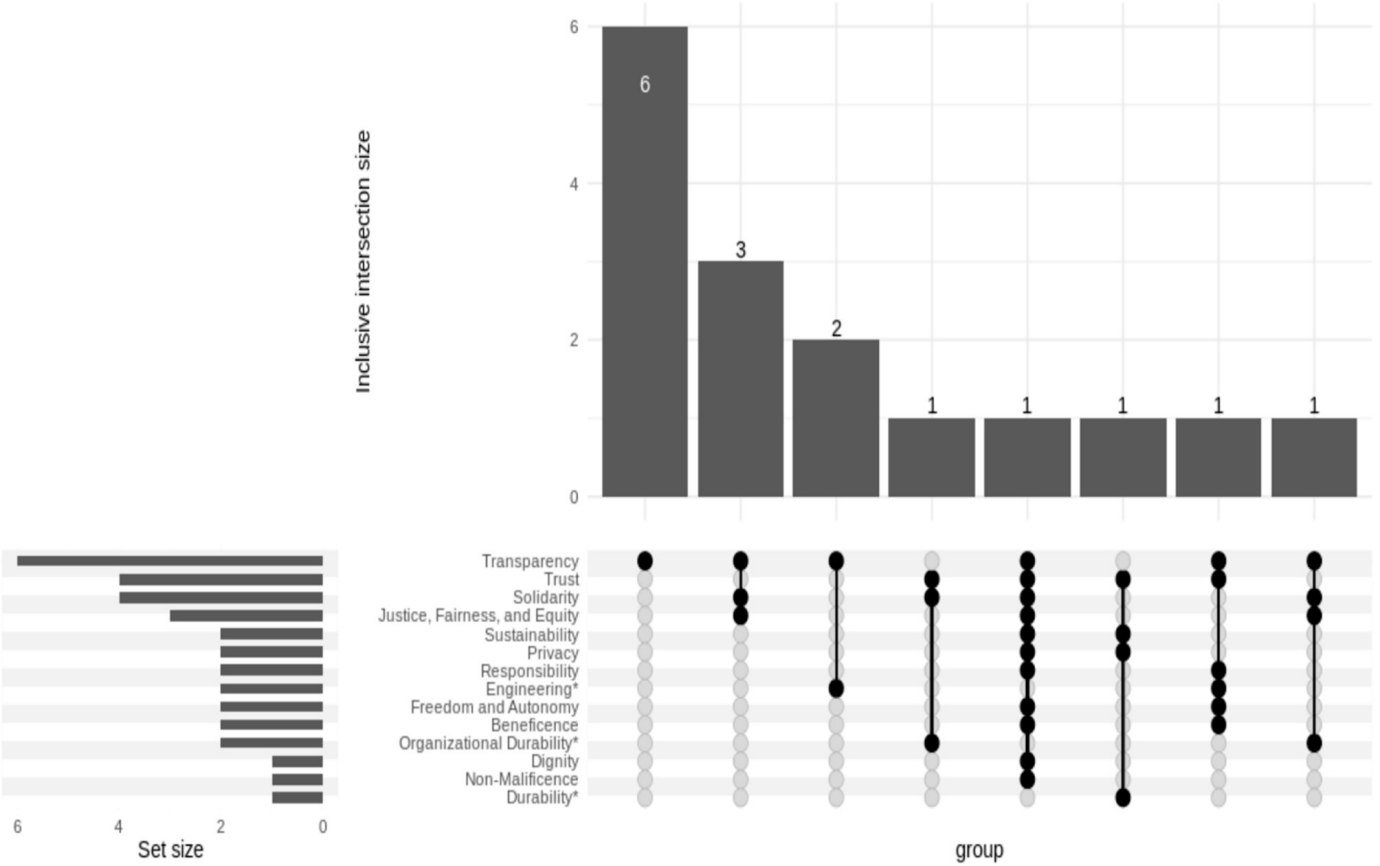
Common core of values. ^*^Denotes an extension to the core categorization developed in Jobin et al. (2019).

The value of *transparency* acts as a least-common-denominator across reports (as shown in Figure 2). However, transparency never appeared alone and was most often included with social equity and solidarity or inclusion. These values are distinct, and some, such as privacy and transparency, are in direct tension.

A dramatic expression of science's dependency on the values embedded in the knowledge ecosystem is the “reproducibility crisis” that has emerged at the interface of science practice and science communication (NASEM-BCBSS, 2019). Reproducibility is essentially a function of transparent scientific information management (Freese and King, 2018), contributing to meta-science, which furthers the values of equity and inclusion as much as those of interpretability and accountability. Open science enhances scientific reliability and human well-being by increasing access to both the process and the fruits of scientific research.

The values inherent in science practices also include the processes of assigning and rewarding value in research, which are themselves functions of science information management: this is the charge that those developing alternatives to bibliometric indicators should accept. Academic organizations determine the perceived value and impact of scholarly work by allocating attention and resources through promotion and tenure processes, collection decisions, and other recognition systems (Maron et al., 2019). As we have learned with economic growth or productivity measures, mechanistic indicators of success do not necessarily align with social and ethical values. Opaque expert and technical systems can undermine public trust unless the values inherent in their design are explicit and communicated clearly (IEEE Global Initiative et al., 2019).

When the academy delegates governance of the scholarly knowledge ecosystem to economic markets, scholarly communication tends toward economic concentration driven by the profit motives of monopolistic actors (e.g., large publishers) and centered within the global north (Larivière et al., 2015). The result has been an inversion of the potential for equity and democratization afforded by technology, leading instead to a system that is:

“plagued by exclusion; inequity; inefficiency; elitism; increasing costs; lack of interoperability; absence of sustainability and/or durability; promotion of commercial rather than public interests; opacity rather than transparency; hoarding rather than sharing; and myriad barriers at individual and institutional levels to access and participation.” (Altman et al., 2018, p. 5)

The imperative to bring the system under a different values regime requires an explicit and coordinated effort that is generated and expressed through research. The reports here reflect the increasing recognition that these values must also inform information research.

Despite emerging as a “loose, feel-good concept instead of a rigorous framework” (Mehra and Rioux, 2016, p. 3), *social justice* in information science has grown into a core concern in the field. Social justice—“fairness, justness, and equity in behavior and treatment” (Maron et al., 2019, p. 34)—may be operationalized as an absence of pernicious discrimination or barriers to access and participation, or affirmatively as the extension of agency and opportunity to all groups in society. A dearth of diversity in the knowledge creation process (along the lines of nationality, race, disability, or gender) constrains the positive impact of advances in research and engineering (Lepore et al., 2020).

Many vital areas of the scientific evidence base, the legal record, and broader cultural heritage are at substantial risk of disappearing in the foreseeable future. Values of information *durability* must be incorporated into the design of the technical, economic, and legal systems governing information to avoid catastrophic loss (NDSA, 2020). The unequal exposure to the risk of such loss is itself a source of inequity. Durability is also linked to the value of *sustainability*, applying both to impact the global environment (Jobin et al., 2019) and the durability of investments and infrastructure in the system, ensuring continued access and functioning across time and space (Maron et al., 2019).

As the information ecosystem expands to include everyone's personal data, the value of *data agency* has emerged to signify how individuals “ensure their dignity through some form of sovereignty, agency, symmetry, or control regarding their identity and personal data” (IEEE Global Initiative et al., 2019, p. 23). The scale and pervasiveness of information collection and use raises substantial and urgent theoretical, engineering, and design questions about how people, systems, and environments create, access, use, curate, and sustain information.

These questions further implicate the need for core values to govern information research and use: if individuals are to be more than objects in the system of knowledge communication, their interaction within that system requires not only access to information but also its *interpretability* beyond closed networks of researchers in narrow disciplines (Altman et al., 2018; NDSA, 2020). Interpretability of information is a prerequisite for the value of *accountability*, which is required to assess the impacts and values of scholarship. Accountability also depends on transparency, as the metrics for monitoring the workings of the scholarly knowledge ecosystem cannot perform their accountability functions unless the underlying information is produced and disseminated transparently.

## Scholarly Knowledge Ecosystem Research Challenges

Governing large information ecosystems presents a deep and broad set of challenges. Collectively, the reports we review touched on a broad spectrum of research areas—shown in Table 4. These research areas range from developing broad theories of epistemic justice (Altman et al., 2018) to specific questions about the success of university-campus strategies for rights-retention (Maron et al., 2019). This section focuses on those research areas representing grand challenges**—**areas with the potential for broad and lasting impact in the foreseeable future.

**Table 4:** Research areas.

**AI ETHICS**	**OPEN SCI**
(Integrating, aligning, and implementing ethical principles through) public policy, technology governance, and research ethics	Costs and infrastructure
**DIGITAL STEWARDSHIP**	Disciplinary differences
Content preservation at scale	Lack of supportive culture, incentives, and training
Content selection at scale	Privacy, security, and proprietary barriers to sharing
Environmental sustainability of digital collections	Structure of scholarly communications
Information cost and value modeling	**REPRODUCIBILITY**
Stewardship at scale	Barriers in the culture of research
Strengthening the evidence base for digital preservation	Fraud and misconduct
Trust frameworks	Obsolescence of digital artifacts
**EAD**	**SCHOLCOM**
(Designing for) political self-determination and data agency	Assessing implicit and explicit bias
(Designing for) universal human values (well-being)	Building business models to support (mission-aligned) scholarly communications
(Designing for) technical dependability	Creating a broader scholarly communications workforce
**GRAND CHALLENGES**	Creating incentives for participation (in scholarly communications)
(Broadening) participation in the research community	Creating metrics built on value: expanding which values we measure
(Overcoming) restrictions on forms of knowledge	Designing systems that focus on users and audience
Incentives to sustain a (ethical) scholarly knowledge ecosystem	Determining the right scale and scope for (technological) infrastructure (that is organizationally sustainable)
Threats to durability of knowledge	Driving transformation within (academic) libraries
Threats to individual agency	Enacting effective strategies for revisiting copyright
Threats to integrity and trust	Encouraging technological innovation and ongoing development (in academic libraries)
**META RESEARCH**	Enhancing representations within academic libraries
Incentives and norms	Ensuring diversity of collections
Reproducibility	Facilitating access for those with disabilities
Statistical misuse	Intentionally limiting openness and knowledge sharing
Transparency	Investing in community-owned infrastructure
	Managing research data and enhancing discovery
	Retaining and protecting intellectual rights
	Understanding the costs of un(der)recognized and un(der)compensated labor (in scholarly communications)

Altman et al. (2018) covered the broadest set of research areas. It identified six challenges for creating a scholarly knowledge ecosystem to globally extend the “true opportunities to discover, access, share, and create scholarly knowledge” in ways that are democratic in their processes—while creating knowledge that is durable as well as trustworthy. These imperatives shape the research problems we face. Such an ecosystem requires expanding *participation* beyond the global minority that dominates knowledge production and dissemination. It must broaden the *forms of knowledge* produced and controlled within the ecosystem, including, for example, oral traditions and other ways of knowing. The ecosystem must be built on a foundation of *integrity and trust*, which allows for the review and dissemination of growing quantities of information in an increasingly politicized climate. With the exponential expansion of scientific knowledge and digital media containing the traces of human life and behavior, problems of the *durability of knowledge*, and the inequities therein, are of growing importance. Opacity in the generation, interpretation, and use of scientific knowledge and data collection, and the complex algorithms that put them to use, deepens the challenge to maintain *individual agency* in the ecosystem. Problems of privacy, safety, and control, intersect with diverse norms regarding access and use of information. Finally, innovations and improvements to the ecosystem must incorporate *incentives for sustainability* so that they do not revert to less equitable or democratic processes.^7^

**Figure 3:**
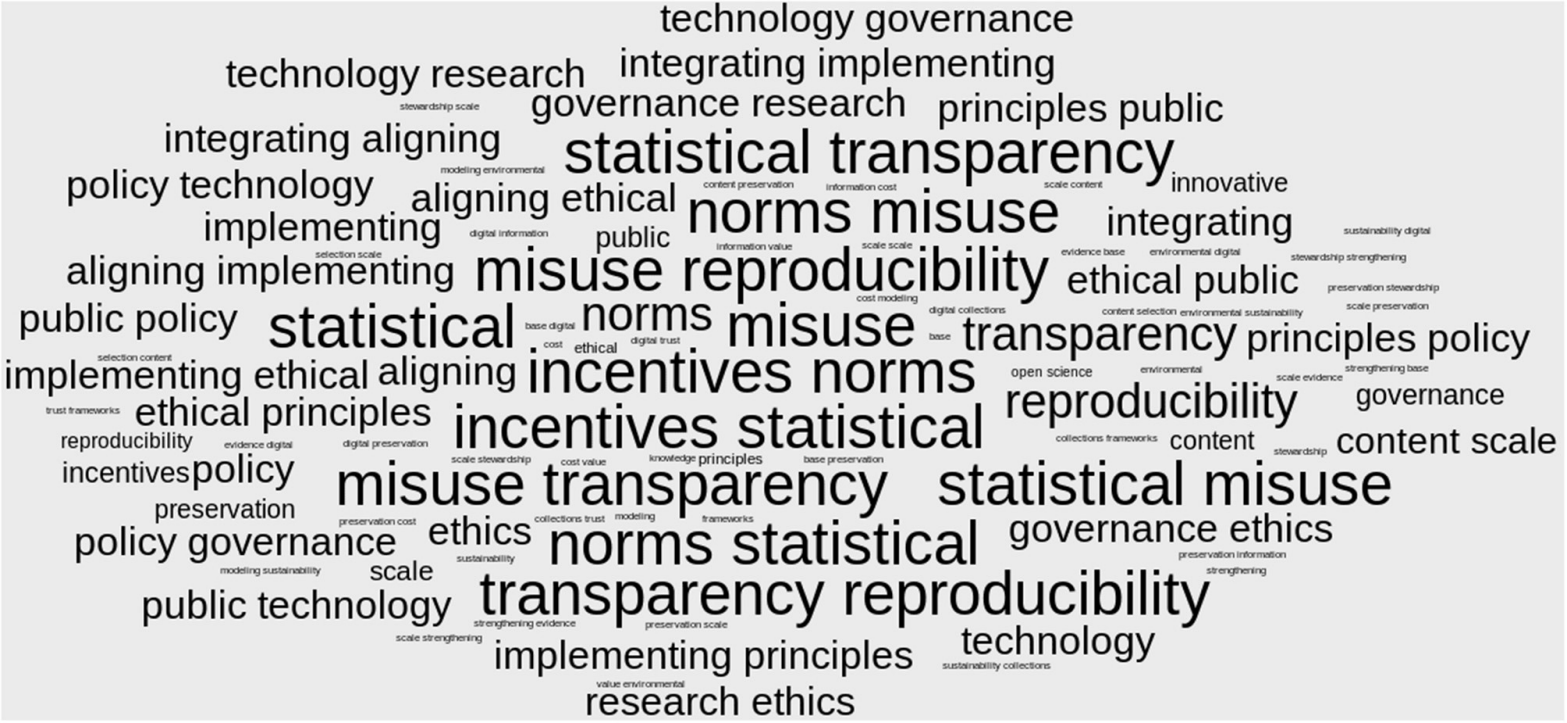
Research problems.

We draw from the frameworks of all the reports to identify several themes for information research. Figure 3 highlights common themes using a term-cloud visualization summarizing research areas and research questions.^8^ The figure shows the importance that the documents place on the values discussed above and the importance of governance, technology, policy, norms, incentives, statistical reproducibility, transparency, and misuse.

For illustration, we focus on several exemplar proposals that reflect these themes. IEEE Global Initiative et al. (2019) asks how “the legal status of complex autonomous and intelligent systems” relates to questions of liability for the harms such systems might cause. This question represents a challenge for law and for ethical AI policy, as Jobin et al. (2019) outlined. Maron et al. (2019) raise questions about how cultural heritage communities limiting access to their knowledge while also making it accessible according to community standards poses additional problems for AI-using companies and the laws that might govern them. There is a complex interaction of stakeholders at the intersections of law, ethics, technology, and information science, and a research agenda to address these challenges will require interdisciplinary effort across institutional domains.

Consider the Grand Challenge's call for research into the determinants of engagement and participation in the scholarly knowledge ecosystem. Understanding those drivers requires consideration of a question raised by Maron et al. (2019) regarding the costs of labor required for open-source infrastructure projects, including the potentially inequitable distribution of unpaid labor in distributed collaborations. Similarly, NASEM–BRDI (2018) and NDSA (2020) delineate the basic and applied research necessary to develop both the institutional and technical infrastructure of stewardship, which would enable the goal of long-term durability of open access to knowledge. Finally, NASEM-BCBSS (2019) and Hardwicke et al. (2020) together characterize the range of research needed to systematically evaluate and improve the trustworthiness of scholarly and scientific communications.

The reports taken as a collection underscore the importance of these challenges and the potential impact that solving them can have far beyond the academy. For example, the NDSA 2020 report clarifies that resolving questions of predicting the long-term value of information and ensuring its durability and sustainability are critical for the scientific evidence-base and for preserving cultural heritage and maintaining the public record for historical government, and for legal purposes. Further, IEEE Global Initiative et al. (2019) and Jobin et al. (2019) demonstrate the ubiquitous need for research into effectively embedding ethical principles into information systems design and practice. Moreover, the IEEE report highlights the need for trustworthy information systems in all sectors of society.

## Commonalities Across the Recommended Solution Approaches to Core Scientific Questions

The previous section demonstrates that strengthening scientific knowledge's epistemological reliability and social equity implicates a broad range of research questions. We argue that despite this breadth, three common themes emerge from the solution approaches in these reports: the need for multi-sectoral cooperation and coordination; the need for mixed methods analysis at multiple levels; and the need for interdisciplinary collaboration.

### Cooperate Across Sectors to Intervene and Measure at Scale

As these reports reiterate, information increasingly “lives in the cloud.”^9^ Almost everyone who creates or uses information, scholars included, relies on information platforms at some point of the information lifecycle (e.g., search, access, publication). Further, researchers and scholars are generally neither the owners of, nor the most influential stakeholder in, the platforms that they use. Even niche platforms, such as online journal discovery systems designed specifically for dedicated scholarly use and used primarily by scholars, are often created and run by for-profit companies and (directly or indirectly) subsidized and constrained by government-sector funders (and non-profit research foundations).

A key implication of this change is that information researchers must develop the capacity to work within or through these platforms to understand information's effective properties, our interactions with these, the behaviors of information systems, and the implications of such properties, interactions, and behaviors for knowledge ecosystems. Moreover, scholars and scientists must be in dialogue with platform stakeholders to develop the basic research needed to embed human values into information platforms, to understand the needs of the practice, and to evaluate both.

### Employ a Full Range of Methodologies Capable of Measuring Outcomes at Multiple Levels

Many of the most urgent and essential problems highlighted through this review require solutions at the ecosystem (macro-) level.^10^ In other words, effective solutions must be implementable at scale and be self-sustaining once implemented. A key implication is that both alternative metrics and vastly greater access to quantitative data from and about the performance of the scholarly ecosystem are required.^11^

### Engage Interdisciplinary Teams to Approach Ecosystem-Level Theory and Design Problems

Selecting, adapting, and employing methods capable of reliable ecosystem-level analysis will require drawing on the experience of multiple disciplines.^12^ Successful approaches to ecosystem-level problems will, at minimum, require the exchange and translation of methods, tools, and findings between research communities. Moreover, many of the problems outlined above are inherently interdisciplinary and multisectoral—and successful solutions are likely to combine insights from theory, method, and practice from information- and computer- science, social- and behavioral- science, and from law and policy scholarship.

These three implications reflect broad areas of agreement across these reports regarding necessary conditions for approaching the fundamental scientific research questions about the scholarly knowledge ecosystem in general. Of course these three conditions are necessary, but far from sufficient—and only scratch the surface of what will be needed to restructure the ecosystem. Developing a comprehensive proposal for such a restructuring is a much larger project—even if the individual scientific questions we summarize above were to be substantially answered. For details on promising approaches to the individual areas summarized in Table 4 see the respective reports, and especially (Altman et al., 2018; Hardwicke et al., 2020; NDSA, 2020).

Moreover, the development of a blueprint to effectively restructure the scholarly ecosystem will require addressing a range of issues. These include the development of effective science practices; effective advocacy in favor or an improved scholarly ecosystem; the development of model information policies and standards (e.g., with respect to licensing, or formats); the construction and operation of information infrastructure; effective education and training; and processes for allocating research funding in alignment with a better functioning ecosystem. Most of the reports discuss above recognize that these issues are critical to any future successful restructuring, and some—especially (Altman et al., 2018; NASEM–BRDI, 2018; Maron et al., 2019; NASEM-BCBSS, 2019)—suggest specific paths forward.

Although the function of this review is to characterize the core scientific challenges to understanding the scholarly ecosystem necessary for a restructuring. We note that there is a growing consensus, as reflected by these reports, around a number of operational principles, practices, and infrastructure that many believe necessary for a positive restructuring of the scholarly knowledge ecosystem. The most broadly recognized examples of these include the FAIR principles for scientific data management (Wilkinson et al., 2016), the TOP guidelines for journal transparency and openness (Nosek et al., 2015), arXiv and the increasingly robust infrastructure for preprints (McKiernan, 2000; Fraser et al., 2020), and the expansion of the infrastructure for data archiving, citation, and discovery (King, 2011; Cousijn et al., 2018; NASEM-BCBSS, 2019; NDSA, 2020) that has been critical to science for over 60 years.

## Summary

Since its inception, the field of information has been a leader in understanding how information is discovered, produced, and accessed. It is now critical to answer these questions as applied to the conduct of research and scholarship itself.

Over the last three decades, the information ecosystem has changed dramatically. The pace of information collection and dissemination has broadened; the forms of scientific information and systems for managing them have become more complex, and the stakeholders and participants in information production and use have vastly expanded. This expansion and acceleration have placed great stress on the system's reliability and heightened internal and external attention to inequities in participation and impact of scientific research and communication.

More recently, the practices and infrastructure for disseminating and curating scholarly knowledge have also begun to change. For example, infrastructure for sharing communications in progress (see, e.g., in preprints, or through alternative forms of publications) is now common in many fields, as is infrastructure to share data for replication and reuse.

These changes present challenges and opportunities for the field of information. While the field's traditional scope of study has broadened from a focus on individual people, specific technologies, and interactions with specific information objects (Marchionini, 2008) to a focus on more general information curation and interaction lifecycles, theories and methods for evaluating and designing information ecologies remain rare (Tang et al., 2021). Further, information research has yet to broadly incorporate approaches from other disciplines to conduct large-scale ecological evaluations or systematically engage with stakeholders in other sectors of society to design and implement broadly-used information platforms. Moreover, while there has been increased interest in the LIS field in social justice, the field lacks systematic frameworks for designing and evaluating systems to promote this value (Mehra and Rioux, 2016).

For scholarship to be epistemologically reliable, policy-relevant, and socially equitable, the systems for producing, disseminating, and sustaining scientific information must be re-theorized, reevaluated, and redesigned. Because of their broad and diverse disciplinary background, information researchers and schools could have an advantage in convening and catalyzing effective research. The field of information science can make outstanding contributions by thoughtful engagement in multidisciplinary, multisectoral, and multimethod research focused on values-aware approaches to information-ecology scale problems.

Thus reimagined and reengineered through interdisciplinary and multisectoral collaborations, the scientific information ecosystem can support enacting evidence-based change in service of human values. With such efforts, we could ameliorate many of the informational problems that are now pervasive in society: from search engine bias to fake news to improving the conditions of life in the global south.

## Author Contributions

The authors describe contributions to the paper using a standard taxonomy (Allen et al., 2014). Both authors collaborated in creating the first draft of the manuscript, primarily responsible for redrafting the manuscript in its current form, contributed to review and revision, contributed to the article's conception (including core ideas, analytical framework, and statement of research questions), and contributed to the project administration and to the writing process through direct writing, critical reviewing, and commentary. Both authors take equal responsibility for the article in its current form.

## Funding

This research was supported by MIT Libraries Open Access Fund.

## Conflict of Interest

The authors declare that the research was conducted in the absence of any commercial or financial relationships that could be construed as a potential conflict of interest.

## Publisher's Note

All claims expressed in this article are solely those of the authors and do not necessarily represent those of their affiliated organizations, or those of the publisher, the editors and the reviewers. Any product that may be evaluated in this article, or claim that may be made by its manufacturer, is not guaranteed or endorsed by the publisher.

## Footnotes

^1^Throughout this paper, we follow Altman et al. (2018) in using the terms “scholarship,” “scholarly record,” “evidence base,” and “scholarly knowledge ecosystem” broadly. These denote (respectively), communities and methods of systematic inquiry aimed at contributing to new generalizable knowledge; all of the informational outputs of that system (including those outputs commonly referred to as “scholarly communications”); the domains of evidence that are used by these communities and methods to support knowledge claims (including quantitative measures, qualitative descriptions, and texts); and the set of stakeholders, laws, policies, economic markets, organizational designs, norms, technical infrastructure, and educational systems that strongly and directly affect the scholarly record and evidence base, and/or are strongly and directly affected by it (which encompasses the system of scholarly communication, and the processes generated by this system).

^2^In order to create a review that spanned multiple disciplines while maintaining concision and lasting relevance we deliberately concentrate the focus of the article in three respects: First, we focus on enduring research challenges rather than on shorter-lived research challenges (e.g., with a time horizon of under a decade). Second, we focus on fundamental challenges to scientific understanding (theorizing, inference, and measurement) rather than on cognate challenges to scholarly practice such as the developing of infrastructure, education, standardization of practice, and the mobilization and coordination of efforts within and across specific stakeholders. Third, we limit discussion of solutions to these problems to describing the contours of broadly applicable approaches—rather than recapitulate the plethora of domain and problem-specific approaches covered in the references cited.

^3^For prominent examples of nanopublication, overlay journals, preprint servers and massive dynamic community databases see (respectively) (Lintott et al., 2008; Groth et al., 2010; Bornmann and Leydesdorff, 2013; Fraser et al., 2020).

^4^For replication purposes, this database and the code for all figures and tables, are available through GitHub https://doi.org/10.7910/DVN/DJB8XI and will be archived in dataverse before publication, and this footnote will be updated to include a formal data citation.

^5^Almost all of the reports stated these values explicitly and argued for their necessity in the design and practice of science. The one exception is (Hardwicke et al., 2020)—which references core values and weaves them into the structure of its discussion—but does not argue explicitly for them.

^6^This set of ethical values constitute ethical principles *for* scientific information and its use. This should be distinguished from research programs such as (Fricker, 2007; Floridi, 2013) who propose ethics *of* information—rules that are inherently normative to information, e.g., Floridi's principle that “entropy ought to be prevented in the infosphere.”

^7^Any enumeration of grand challenge problems inevitably tends to the schematic. This ambitious map of challenges, intended to drive research priorities, has the benefit of reflecting the input of a diverse range of participants. Like the other reports in our review, Altman et al. (2018) lists many contributors (14) from among even more (37) workshop participants, and followed by a round of public commentary. Such collaboration will also be required to integrate responses, as these challenges intertwine at their boundaries. Thus, successful interventions to change the ecosystem at scale will require working in multiple, overlapping problem areas. Notwithstanding, these problems are capacious enough that any one of them could be studied separately and prioritized differently by different stakeholders.

^8^Figure 3 is based on terms generated through skip n-gram analysis and ranked by their importance within each document relative to the entire corpus. Specifically, the figure uses TIF^*^DF (term frequency by inverse document frequency) to select and scale 2 by 1 skip-n-grams extracted from the entire corpus after minimal stop-word removal. This results in emphasizing pairs of words such as “transparency reproducibility” that do not appear in most documents overall, but appear together frequently within some documents.

^9^Specifically, see NASEM-BIRDI (2018, chapters one and two), Lazer et al. (2009), and NDSA et al. (2020, sections 1.1, 4.1, and 5.2).

^10^Ecosystem-level analysis and interventions are an explicit and central theme of Altman et al. (2018), NASEM–BRDI (2018), Maron et al. (2019) and Hardwicke et al. (2020) refer primarily to ecosystems implicitly in emphasizing throughout on the global impacts of and participation in interconnected networks of scholarship. NDSA (2020) explicitly addresses ecosystem issues through discussion of shared technical infrastructure and practices (see section 4.1) and implicitly through multi-organizational coordination to steward shared content and promote good practice.

^11^Metrics are a running theme of IEEE Global Initiative et al. (2019)—especially the ubiquitous need for open quantitative metrics of system effectiveness and impact, and the need for new (alternative) metrics to capture impacts of engineered systems on human well-being that are currently unmeasured. Altman et al. (2018, see, e.g., section 2) notes the severe limitations of the current evidence base and metrics for evaluating scholarship and the functioning of the scholarly ecosystem. Jobin et al. (2019, p. 389) also note the importance of establishing a public evidence base to evaluate and govern ethical AI use. Similarly, NDSA et al. (2020, section 5.2) emphasize the need to develop a shared evidence base to evaluate the state of information stewardship. Maron et al. (2019) call for new (alternative) metrics and systems of evaluation for scholarly output and contents as a central concern for the future of scholarship (p. 11–13, 16–20). NASEM–BRDI (2018), NASEM-BCBSS (2019), and Hardwicke et al. (2020) emphasize the urgent need for evidential transparency in order to evaluate individual outputs and systemic progress toward scientific openness and reliability—and emphasize broad sharing of data and software code.

^12^IEEE Global Initiative et al. (2019) emphasized interdisciplinary research and education as one of the three core approaches underpinning ethical engineering research and design (pp. 124–129), and identifying the need for interdisciplinary approaches in specific key areas (particularly engineering and well-being, affective computing, science education, and science policy). Altman et al. (2018) emphasize the need for interdisciplinarity to address grand challenge problems, arguing that an improved scholarly knowledge ecosystem “will require exploring a set of interrelated anthropological, behavioral, computational, economic, legal, policy, organizational, sociological, and technological areas.” Maron et al. (2019, sec. 1) call out the need for situating research in the practice and the engagement of those in the information professions. NDSA (2020) argue that solving problems or digital curation and preservation require transdisciplinary (sec. 2.5) approaches and drawing on research from a spectrum of disciplines, including computer science, engineering, and social sciences (sec 5). NASEM-BCBSS (2019) note that reproducibility in science is a problem that applies to all disciplines. While NASEM–BRDI (2018) and Hardwicke et al. (2020) both remark that the body of methods, training, and practices (e.g., meta-science, data science) required for achieving open and reproducible (respectively) science require approaches that are inherently inter-/cross-disciplinary.

## References

[B1] Allen L. Scott J. Brand A. Hlava M. Altman M. (2014). Publishing: credit where credit is due. Nature 508, 312–313. 10.1038/508312a 24745070

[B2] Altman M. Bourg C. Cohen P. N. Choudhury S. Henry C. Kriegsman S.. (2018). “A grand challenges-based research agenda for scholarly communication and information science,” in MIT Grand Challenge Participation Platform, Cambridge, MA

[B3] Björnberg K. E. Karlsson M. Gilek M. Hansson S. O. (2017). Climate and environmental science denial: a review of the scientific literature published in 1990–2015. J. Clean. Prod. 167, 229–241. 10.1016/j.jclepro.2017.08.066

[B4] Bornmann L. Leydesdorff L. (2013). The validation of (advanced) bibliometric indicators through peer assessments: a comparative study using data from InCites and F1000. J. Inform. 7, 286–291. 10.1016/j.joi.2012.12.003

[B5] Bornmann L. Mutz R. (2015). Growth rates of modern science: a bibliometric analysis based on the number of publications and cited references. J. Assoc. Inform. Sci. Technol. 66, 2215–2222. 10.1002/asi.2332925855820

[B6] Callaway E.. (2020). Will the pandemic permanently alter scientific publishing? Nature 582, 167–169. 10.1038/d41586-020-01520-4 32504015

[B7] Camerer C. F. Dreber A. Holzmeister F. Ho T.-H. Huber J. Johannesson M.. (2018). Evaluating the replicability of social science experiments in Nature and Science between 2010 and 2015. Nat. Human Behav. 2, 637–644. 10.1038/s41562-018-0399-z 31346273

[B8] Cousijn H. Kenall A. Ganley E. Harrison M. Kernohan D. Lemberger T.. (2018). A data citation roadmap for scientific publishers. Sci. Data 5, 1–11. 10.1038/sdata.2018.259 30457573 PMC6244190

[B9] Floridi L.. (2013). The Ethics of Information. Oxford: Oxford University Press.

[B10] Fraser N. Brierley L. Dey G. Polka J. K. Pálfy M. Coates J. A. (2020). Preprinting a pandemic: the role of preprints in the COVID-19 pandemic. BioRxiv 2020.05.22.111294. 10.1101/2020.05.22.11129433798194

[B11] Freese J. King M. M. (2018). Institutionalizing transparency. Socius 4:2378023117739216. 10.1177/2378023117739216

[B12] Fricker M.. (2007). Epistemic Injustice: Power and the Ethics of Knowing. Oxford: Oxford University Press.

[B13] Groth P. Gibson A. Velterop J. (2010). The anatomy of a nanopublication. Inform. Serv. Use. 30, 51–56. 10.3233/ISU-2010-0613

[B14] Hardwicke T. E. Serghiou S. Janiaud P. Danchev V. Crüwell S. Goodman S. N.. (2020). Calibrating the scientific ecosystem through meta-research. Annu. Rev. Stat. Appl. 7, 11–37. 10.1146/annurev-statistics-031219-04110422096103

[B15] IEEE Global Initiative Chatila, R. Havens J. C. (2019). “The IEEE Global Initiative on Ethics of Autonomous and Intelligent Systems,” in Robotics and Well-Being, eds M. I. Aldinhas Ferreira, J. Silva Sequeira, G. Singh Virk, M. O. Tokhi, and E. E. Kadar (Springer International Publishing), 11–16.

[B16] Jobin A. Ienca M. Vayena E. (2019). The global landscape of AI ethics guidelines. Nat. Mach. Intell. 1, 389–399. 10.1038/s42256-019-0088-2

[B17] King G.. (2011). Ensuring the data-rich future of the social sciences. Science 331, 719–721. 10.1126/science.1197872 21311013

[B18] Larivière V. Haustein S. Mongeon P. (2015). The oligopoly of academic publishers in the digital era. PLoS ONE 10:e0127502. 10.1371/journal.pone.0127502 26061978 PMC4465327

[B19] Lazer D. Pentland A. Adamic L. Aral S. Barabasi A.-L. Brewer D.. (2009). Social science: computational social science. Science 323, 721–723. 10.1126/science.1167742 19197046 PMC2745217

[B20] Leek J. T. Jager L. R. (2017). Is most published research really false? Annu. Rev. Stat. Appl. 4, 109–122. 10.1146/annurev-statistics-060116-054104

[B21] Lepore W. Hall B. L. Tandon R. (2020). The Knowledge for Change Consortium: a decolonising approach to international collaboration in capacity-building in community-based participatory research. Can. J. Dev. Stud. 2020, 1–24. 10.1080/02255189.2020.1838887

[B22] Lintott C. J. Schawinski K. Slosar A. Land K. Bamford S. Thomas D.. (2008). Galaxy Zoo: morphologies derived from visual inspection of galaxies from the Sloan Digital Sky Survey. Month. Notices R. Astron. Soc. 389, 1179–1189. 10.1111/j.1365-2966.2008.13689.x

[B23] Marchionini G.. (2008). Human–information interaction research and development. Library Inf. Sci. Res. 30, 165–174. 10.1016/j.lisr.2008.07.001

[B24] Maron N. Kennison R. Bracke P. Hall N. Gilman I. Malenfant K.. (2019). Open and Equitable Scholarly Communications: Creating a More Inclusive Future. Chicago, IL: Association of College and Research Libraries.

[B25] McKiernan G.. (2000). ArXiv. Org: The Los Alamos National Laboratory e-print server. Int. J. Grey Literat. 1, 127–138. 10.1108/14666180010345564

[B26] Mehra B. Rioux K. (eds.). (2016). Progressive Community Action: Critical Theory and Social Justice in Library and Information Science. Sacramento, CA: Library Juice Press.

[B27] NASEM-BCBSS Board on Behavioral, Cognitive, and Sensory Sciences, Committee on National Statistics, Division of Behavioral and Social Sciences and Education, Nuclear and Radiation Studies Board, Division on Earth and Life Studies, Board on Mathematical Sciences and Analytics. (2019). Reproducibility and Replicability in Science. Washington, DC: National Academies Press.

[B28] NASEM–BRDI Board on Research Data and Information, Policy and Global Affairs and National Academies of Sciences Engineering and Medicine. (2018). Open Science by Design: Realizing a Vision for 21st Century Research. Washington, DC: National Academies Press, p. 25116 30212065

[B29] National Research Council (2009). Strengthening Forensic Science in the United States: A Path Forward. Washinton, DC: National Research Council.

[B30] NDSA National Digitial Stewardship Alliance, and National Agenda Working Group. (2020). 2020 NDSA Agenda. Washinton, DC: NDSA.

[B31] Nosek B. A. Alter G. Banks G. C. Borsboom D. Bowman S. D. Breckler S. J.. (2015). Promoting an open research culture. Science 348, 1422–1425. 10.1126/science.aab2374 26113702 PMC4550299

[B32] Obermeyer Z. Powers B. Vogeli C. Mullainathan S. (2019). Dissecting racial bias in an algorithm used to manage the health of populations. Science 366, 447–453. 10.1126/science.aax2342 31649194

[B33] Sun T. Gaut A. Tang S. Huang Y. ElSherief M. Zhao J.. (2019). Mitigating gender bias in natural language processing: literature review. ArXiv:1906.08976 [Cs]. http://arxiv.org/abs/1906.08976

[B34] Tang R. Mehra B. Du J. T. Zhao Y. (Chris). (2021). Framing a discussion on paradigm shift(s) in the field of information. J. Assoc. Inf. Sci. Technol. 72, 253–258. 10.1002/asi.2440425855820

[B35] Tavernier W.. (2020). COVID-19 demonstrates the value of open access: what happens next? College Res. Libr. News 81:226. 10.5860/crln.81.5.22617723383

[B36] Tingley K.. (2020, April 21). Coronavirus Is Forcing Medical Research to Speed Up. The New York Times. https://www.nytimes.com/2020/04/21/magazine/coronavirus-scientific-journals-research.html

[B37] Wilkinson M. D. Dumontier M. Aalbersberg I. J. Appleton G. Axton M.. (2016). The FAIR Guiding Principles for scientific data management and stewardship. Sci. Data 3:160018. 10.1038/sdata.2016.18 26978244 PMC4792175

